# Variation in cyanogenic compounds concentration within a *Heliconius* butterfly community: does mimicry explain everything?

**DOI:** 10.1186/s12862-016-0843-5

**Published:** 2016-12-15

**Authors:** Mónica Arias, Aimilia Meichanetzoglou, Marianne Elias, Neil Rosser, Donna Lisa de-Silva, Bastien Nay, Violaine Llaurens

**Affiliations:** 1 Institut Systématique, Evolution, Biodiversité, UMR 7205 MNHN-CNRS-EPHE-UPMC- Sorbonne Universités, Muséum National d’Histoire Naturelle, Bâtiment d’entomologie, CP050, 57 rue Cuvier, 75005 Paris, France; 2 Unité Molécules de Communication et Adaptation des Micro-organismes, UMR 7245 MNHN-CNRS, Sorbonne Universités, Muséum National d’Histoire Naturelle and CNRS, 57 rue Cuvier, CP 54, 75005 Paris, France; 3 Department of Biology, University of York, Heslington, York, YO10 5DD UK

## Abstract

**Background:**

Aposematic species advertise their unpalatability using warning signals such as striking coloration. Given that predators need to sample aposematic prey to learn that they are unprofitable, prey with similar warning signals share the cost of predator learning. This reduction in predation risk drives evolutionary convergence of warning signals among chemically defended prey (Müllerian mimicry). Whether such warning signal convergence is associated to similar defence levels among co-mimics is still an open question that has rarely been tested in wild populations. We quantified variation in cyanide-based (CN) chemical protection in wild caught individuals of eight aposematic *Heliconius* butterfly species belonging to four sympatric mimicry rings. We then tested for correlations between chemical protection and ecological species-specific traits.

**Results:**

We report significant differences in CN concentrations both within and between sympatric species, even when accounting for the phylogeny, and within and between mimicry rings, even after considering inter-specific variation. We found significant correlations between CN concentration and both hostplant specialization and gregarious behaviour in adults and larvae. However, differences in CN concentrations were not significantly linked to mimicry ring abundance, although the two most toxic species did belong to the rarest mimicry ring.

**Conclusions:**

Our results suggest that mimicry can explain the variation in the levels of chemical defence to a certain extent, although other ecological factors are also relevant to the evolution of such variability.

**Electronic supplementary material:**

The online version of this article (doi:10.1186/s12862-016-0843-5) contains supplementary material, which is available to authorized users.

## Background

Toxic species displaying bright colour patterns that advertise their unpalatability to predators are said to be aposematic [1, 2]. Although the association between warning coloration and distastefulness can rely on predators’ innate biases [3], they usually need several sampling events to learn it [4–7]. This predation pressure promotes evolutionary convergence in colour patterns between chemically protected species living in sympatry, because species that share a common warning signal share the cost of predator learning. This association is known as Müllerian mimicry [8], and different species that exhibit the same warning signal are said to form “mimicry rings”. Müllerian mimicry has been observed in various unpalatable organisms such as insects [9, 10] and amphibians [11]. Similar protection between Müllerian co-mimics has been classically assumed in theoretical approaches as it is modelled as a strictly mutualistic interaction. However, when co-mimetic species exhibit differences in defence levels, less protected mimics might dilute the protection of a given warning signal, acting in a semi-parasitic manner (i.e., quasi-batesian mimicry [12]). Uneven defences within mimicry ring can then promote warning signal shift in the most toxic species toward better-protected mimicry rings [13]. Such processes might homogenize defence levels among Müllerian mimics but empirical studies estimating defence variations within natural communities are still lacking. Species that are considered Müllerian co-mimics can rely on drastically different chemical compounds [14], and chemical defences can be either sequestered from diet [15–18] or neo-synthesized [19, 20]. Consequently, co-mimics are not always equally unpalatable, with levels of chemical protection varying from very similar to very uneven, as reported in some mimetic butterflies [21, 22] and frogs [23, 24]. Even within species, individuals are not equally protected. In extreme cases, this intraspecific variation includes palatable individuals within protected species; an interaction known as automimicry [25]. Automimics thus benefit from the unpalatability of their co-mimics, without investing in chemical protection themselves. This variation in defence levels between mimics can be linked to several ecological factors.

Factors associated to the amount of prey encountered by predators (abundance) and how memorable such encounters are (enhanced by behaviours such as aggregation, for instance [4, 26] but for contrasting evidence see [27]) might be correlated with different defence levels. Moreover, when defences are sequestered, the efficiency in the use of the available resources (larger for specialist than for generalist feeders, for example [20, 28]) is also likely to play an important role in the evolution of chemical defences. Additionally, differences in the resource use between sexes associated to their relative vulnerability intrinsic to their specific ecological roles [23], need also to be considered when studying differences in chemical protection. All these factors are correlated, and might have a joint effect on defence level variation and warning signal convergence. Here we investigate the effect of those multifarious ecological traits in chemical protection variation.

Here, we focus on Neotropical *Heliconius* butterflies, which exhibit several outstanding examples of mimetic convergence between distantly related species both within [29] and outside the genus [30]. *Heliconius* butterflies contain toxic cyanogenic glucosides obtained from their *Passiflora* host plants during larval feeding [30, 31], and also through *de novo* synthesis as larvae and adults [16, 20]. Although all *Heliconius* have similar chemical compounds, they participate in a number of different sympatric mimicry rings, allowing investigation of variations in toxicity both within and between mimicry rings in a single community. Several previous studies have investigated toxicity (i.e., chemical compounds) and unpalatability (i.e., predators behaviour) variations in *Heliconius* butterflies. Studies of natural and experienced predators found differences in rejection behaviour towards several *Heliconius* species [32, 33]. However, no attempts were made to disentangle the visual and chemical components of aposematic prey. Chemical analyses have also revealed differences in the concentration of cyanogenic compounds in *Heliconius* butterflies, highlighting in particular the apparent association between the specialisation of *Heliconius sara* on the larval host-plant *Passiflora auriculata* and a significant increase in toxicity compared to generalist species [20, 34, 35]. However, most butterflies used in these studies were captive-bred, and in several cases were not reared on their natural host plant species. The variations in toxicity in natural populations, which are the products of multifarious ecological factors, have yet to be investigated.

By comparing toxicity in *Heliconius* species sharing warning signals but with contrasting abundances, and distinct behavioural (i.e., larval aggregation, communal roosting) and physiological traits (i.e., host-plant specialisation, capacity to synthesise cyanogenic glucosides), we test for associations between those different traits and chemical defence levels. We measured cyanide levels in wild caught individuals belonging to eight different sympatric *Heliconius* species, aiming to 1) quantify the variation of cyanide concentration within and between sympatric protected species. We also aim to test whether 2) co-mimetic species have similar levels of toxicity, 3) coexisting mimicry rings have different toxicity levels, and 4) differences in toxicity are correlated with a) the local abundance of the mimicry ring, b) sex, and c) life history traits such as communal roosting, larvae gregariousness and dietary specialisation.

## Methods

### Sample collection

**Fig. 1:**
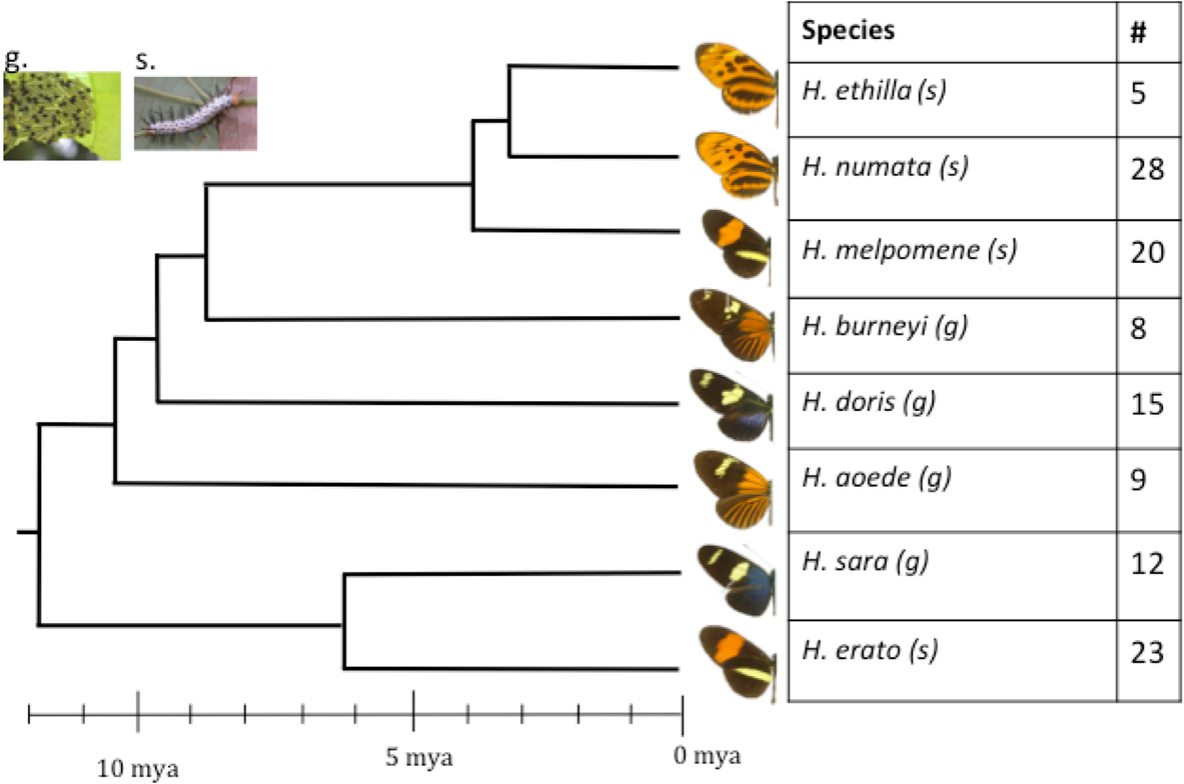
Phylogenetic relationships of the 8 species used in this study and number of samples per species. Phylogeny dated according to Kozak et al. [29]. The two main types of larvae are shown in the top left; g) brown/green and gregarious and s) white and solitary, and are associated to each species (in parenthesis)

Butterflies were collected in natural populations in the vicinity of Tarapoto (San Martin department, Peru) in September 2014. Butterflies’ head, thorax and abdomen were stored in methanol (wings were discarded because the head and thorax of *Heliconius* butterflies usually contain the highest CN concentrations [36]). Our sample included 8 *Heliconius* species belonging to 4 different mimicry rings, thus encompassing all the local *Heliconius* mimicry rings (see Fig. 1): *H. numata* (*n* = 28, 7 females, 20 males, 1 not registered sex (NRS)) and *H. ethilla* (*n* = 5, 2 females, 3 males) (*tiger* ring)*, H. erato* (*n* = 23, 5 females, 17 males, 1 NRS) and *H. melpomene* (*n* = 20, 6 females, 13 males, 1 NRSS) (*postman* ring), *H. aoede* (*n* = 9, 4 females, 5 males) and *H. burneyi* (*n* = 8, 1 female, 7 males) (*dennis-rayed* ring)*, H. sara* (*n* = 12, 2 females, 10 males) and *H. doris* (*n* = 15, 9 females, 6 males) (*blue/yellow* ring). While there is evidence that *Heliconius* mimicry rings are segregated by habitat to some degree [37], all of these butterflies occur in the same broadly defined community, and can be seen flying together.

### Cyanide extraction

Methanol was removed from the collection tube using a vacuum concentrator (Savant AES 1010 Speedvac® system: ThermoFisher Scientific France). Dried butterfly bodies were then ground to a fine powder using liquid nitrogen in a mortar and the powdered tissue was weighed. First we extracted all cyanogenic glucosides (CGs) by adding 0.1 M H_3_PO_4_ (4 mL, also used to rinse the butterfly collection flask) and stirring the mixture at room temperature for 1 h. The mixture was then filtered using 7 mm diameter glass pipettes and cotton. To hydrolyse the cyanogenic compounds by cleaving the glycosidic bond and releasing the cyanohydrin aglycone (Fig. 3), 2 mL aliquot of the filtrate was mixed with 2 mL of 5 M H_2_SO_4_, in a tightly capped tube and heated in boiling water (100 °C) for 1 h. The hot solution was cooled in an ice bath and 5 mL of ice-cold 5 M NaOH was added to hydrolyse the cyanohydrin aglycone and trap the liberated cyanide as NaCN. The basic solutions were allowed to stand for 1 h at room temperature to ensure complete reaction.

### Quantification of cyanogenic glucosides by colorimetric analysis

The following method was adapted from Lambert et al. [38]. Aliquots (125 μL) of the final basic solution were poured into three different test tubes containing 0.2 M phosphate buffer (875 μL, pH 6), allowing independent measures of three technical replicates for each biological sample. 0.4 mL of *N*-chlorosuccinimide/succinimide (NCS) oxidizing reagent solution was added to each tube (see Additional file 1 for detailed NCS preparation). These oxidised solutions were then kept at 20–21 °C for 20 min, after which 1.6 mL of pyridine/barbituric acid solution (chromogenic reagent, see Additional file 1 for preparation) was added. After 20 min, when the mixture developed a purple colour, the absorbance of the sample was measured using a spectrophotometer (UVIKON UV 9×3 W, BioServ France) at 580 nm against a blank solution (phosphate buffer + reagents). The NaCN concentration for each sample was then calculated by comparing its absorbance with a calibration curve calculated using solutions of known NaCN concentration, with standard absorbance of each sample on the *x*-axis and known NaCN concentration on the *y-*axis (NaCN range 0.2–18 μg/mL).

### Ecological and life-history traits

**Fig. 2:**
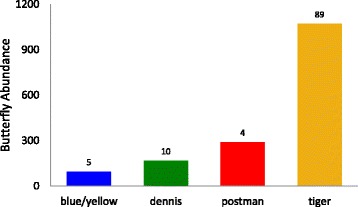
Abundances of butterflies belonging to each mimicry ring in the Peruvian departments of San Martin and Loreto. Samples were collected on January-March 2011, August-December 2011, January-March 2012, September 2014 and from June 2015 to April 2016, and were pooled together. On top of the bars is the number of species belonging to each mimicry ring

Abundance (the total number of individuals in each mimicry ring) was estimated from sampling performed in San Martin and Loreto areas in January-March 2011, August-December 2011, January-March 2012, September 2014 and June 2015 to April 2016 (Fig. 2). We included all protected mimetic species (*Heliconius*, ithomiine butterflies and *Chetone* moths) involved in each of the four mimicry rings (tiger, postman, dennis-rayed and blue/yellow, Additional file 2: Table S1). Data on roosting behaviour, larval gregariousness and host plant use were obtained from the literature [30, 39–42] and are summarized in Table 1.

**Table 1:** Characters proposed as possible explanatories of the toxicity variability found in our study

Species	Mimicry ring	Communal roosting	Gregarious larvae	Host plant specialisation
*H. ethila*	tiger	yes	no	specialist
*H. numata*	tiger	no	no	generalist
*H. doris*	blue/yellow	no	yes	specialist
*H. sara*	blue/yellow	yes	yes	specialist
*H. aoede*	rayed	no	yes	specialist
*H. burneyi*	rayed	no	yes	specialist
*H. melpomene*	postman	yes	no	(semi)specialist
*H. erato*	postman	yes	no	(semi)generalist

### Statistical analyses

To explore whether convergence in colour pattern is associated to the convergence of each of the evaluated traits, we performed a Factor Analysis of Mixed Data (FAMD) using *FAMD* in the R-package *FactoMineR* [43]*.* FAMD performs Principal Component Analyses on continuous variables and Multiple Correlation Analyses on categorical variables, enabling the simultaneous analysis of both kinds of factors. Abundance was excluded given that both species within each mimicry ring share the same abundance, thus, it could work as a grouping factor.

To test whether phylogenetic relationships could account for toxicity variation in our samples we computed 1) Blomberg’s *K* [44] and 2) Pagel’s lambda [45, 46] for toxicity, and testing their significance using *phytools* [47]. In all cases we used a comprehensive phylogeny for the genus *Heliconius* [29] that was pruned to include only the species in our study. We then used *phytools* [47] to test for differences in cyanide concentration between species using a phylogenetic ANOVA and posthoc tests, while controlling for multiple testing (Holm-Bonferroni method). Differences between species were also tested using a Wilcoxon test that does not include the phylogenetic effect. To investigate whether CN concentration is related to life history traits such as feeding specialization, larval gregariousness, roosting behaviour or mimicry ring, while accounting for intraspecific variation and phylogeny, we expanded the method of phylogenetic ANOVA to incorporate a nested structure. “Nested phylogenetic ANOVAs” work as follows: first, we simulated the evolution of CN concentration at the species-level phylogeny 1,000 times (*i. e.* one value μ_sim_i_ for each species *i* for each simulation *sim*), following a Brownian motion and assuming an instantaneous variance of the Brownian motion process equal to the mean squared value of the Phylogenetic Independent Contrasts (PICs) for CN concentration [47–49]. Then, we estimated the observed mean and standard deviation of CN concentration for each species *i*: μ_obs_i_ and σ_obs_i_. Afterwards, for each individual of each species in the original dataset, we sampled a value for CN concentration in a normal distribution centered on μ_sim_i_ and with standard deviation (σ_obs_i_/μ_obs_i_)*μ_sim_i_, with σ_obs_i_/μ_obs_i_ as the coefficient of variation. Having simulated data at the individual level, we subsequently performed a nested ANOVA on each simulation, using as nested each of the factors mentioned above. We recorded F statistics for the different nesting levels in each simulation, and we generated a distribution of those statistics. To test for differences between mimicry rings we tested pairwise comparisons with a *t* test accounting for variation within mimicry ring, and controlling for multiple testing with the Bonferroni method. Finally, we performed a nested ANOVA on the actual dataset, and compared the observed statistics to the distribution of the simulated statistics, to calculate *p*-values. To test for correlations between cyanide concentration and sex, while controlling for phylogeny, we performed a “phylogenetic two-way ANOVA”. We simulated data as for the “Nested phylogenetic ANOVAs”. Differences between sexes in each species were also tested by a Wilcoxon test.

We tested whether mimicry ring abundance and toxicity were correlated after controlling for phylogeny using Phylogenetic Generalized Least Squares (PGLS). To account precisely for the phylogenetic signal in the correlation between toxicity and abundance, we applied the method suggested by Symonds and Blomberg [50]. First, we calculated a linear regression between toxicity and abundance. Then, we estimated the phylogenetic signal on its residuals by calculating Pagel’s lambda [45, 46] using BayesTraits V.2 [51]. The phylogeny was then transformed according to lambda. A linear model was then fitted using PGLS and the transformed phylogeny, with the package *phytools* [47].

## Results

### Variations of toxicity within and among species

**Fig. 3:**
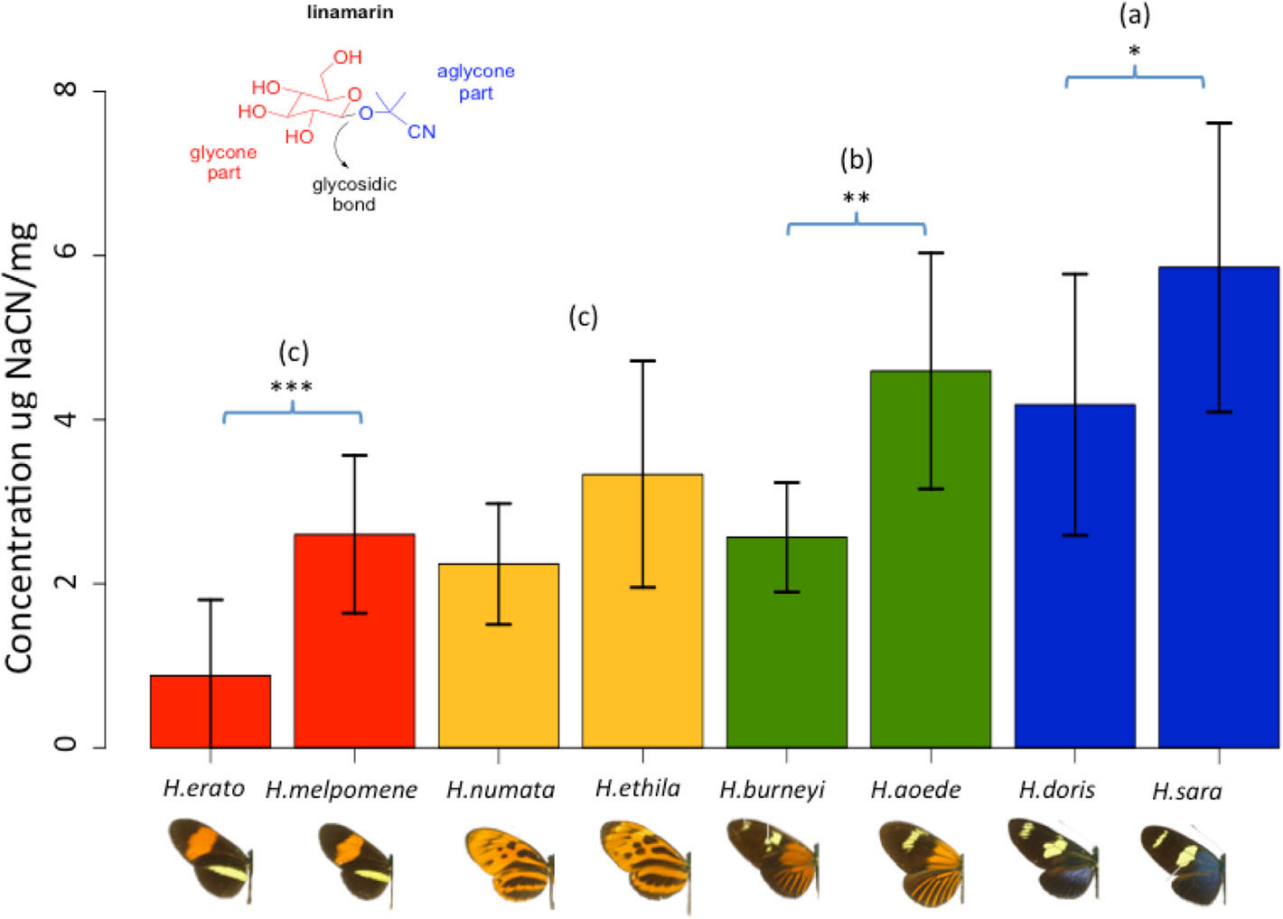
Mean and standard deviation (lines) of the concentration μg of NaCN per mg for each species. *** stands for significant difference with *P* < 0.001, ** for significant difference with *P* <0.01, * for significant difference with *P* < 0.05 between co-mimics. Letters stand for significant differences in the comparisons on the concentration of NaCN between mimicry rings (*p* < 0.05). On the top left corner is an example of a cyanogenic glucoside molecule (linamarin), showing the glycosidic bond between the glycine and the aglycone part that was broken off during hydrolysis

A large variation in cyanide concentration was found among the compared species (Fig. 3, Table 1). For instance, although *H. erato* and *H. sara* are closely related (Fig. 1), the latter has a CN concentration six times higher than the former (Fig. 3). No significant phylogenetic signal was detected on the CN concentration of our samples (Blomberg’s *K* = 0.414, *p* = 0.54 and Pagel’s lambda = 5.07. 10^−05^, *p* = 0.12). However, the high *K* value and small sample size of our study mean that phylogenetic signal cannot be completely discounted. We therefore performed all tests twice, with and without phylogenetic correction.

Females generally displayed a higher cyanide concentration than males (*two-way ANOVA without phylogenetic correction*: sex *df* = 1, *F =* 10.116, *p* = 0.002; sex*species *df* = 7, *F =* 2.753, *p* = 0.012; *with phylogenetic correction:* sex *df* = 1, *F =* 10.116, *p* = 0; sex*species *df* = 7, *F =* 2.753, *p* = 0.012). However, sex differences were non-significant within many species probably due to small sample sizes for females (*Wilcoxon test H. erato W =* 42, *p* = 1; *H. melpomene W =* 46.5, *p =* 0.54*; H. numata W =* 61.5, *p* = 0.66*; H. burneyi W =* 7*, p =* 0.25*; H. aoede W =* 16*, p =* 0.19*; H. ethilla W =* 0, *p* = 0.2*; H. sara W* = 10, *p* = 1). Therefore, the overall significant difference between sexes was mostly driven by *H. doris*, whose females had 1.6 times higher CN concentration than males (*Wilcoxon test W* = 46.5, *p =* 0.025).

### Variations among mimicry rings

We found significant differences in cyanide concentration between mimicry rings, even after accounting for inter-specific variation (*nested phylogenetic ANOVA* mimicry ring (*mr*) *F =* 48.46, *p =* 0; *mr*:*species F =* 13.77, *p =* 0; *nested ANOVA mr df* = 3, *p* < 0.001, *mr*:*sp df* = 4, *p* < 0.001). The most toxic ring was the ‘*blue/yellow*’ mimicry ring, followed by the ‘*dennis-rayed*’ one. The ‘*postman*’ and ‘*tiger*’ mimicry rings were similarly toxic and showed a lower cyanide concentration than the other mimicry rings (Fig. 3). However, we also detected significant differences in cyanide concentration between most pairs of co-mimetic species (Fig. 3, and Table 2).

**Table 2:**
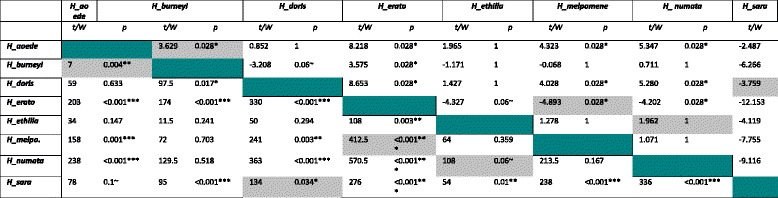
*t* values and posthoc *p* calculations from phylogenetic ANOVA (above diagonal) and Wilcoxon test results for pairwise comparisons between tested species (below diagonal)

In grey cells, comparisons between co-mimic species. *** stands for significant difference with *P* < 0.001, ** for significant difference with *P* <0.01, * for significant difference with *P* < 0.05 and ~ for significant difference with *P* < 0.1

### Correlation with ecological and life-history traits

Species whose adults roost communally were generally less toxic than solitary roosters (*nested phylogenetic ANOVA* roosting *F =* 4.99*, p* = 0.03, roosting:species *F* = 32.58, *p* < 0.001; *nested ANOVA* roosting *df* = 1, *p* = 0.03, roosting:species *df* = 6, *p* < 0.001), in accordance with the expectation that aggregation may enhance warning signal efficiency in poorly defended species. However, species with gregarious larvae were more toxic than species with solitary larvae (*nested phylogenetic ANOVA* gregariousness *F =* 124.92, *p* < 0.001; gregariousness:species *F =* 12.59, *p* < 0.001; *nested ANOVA* gregariousness *df* = 1, *p* < 0.001; gregariousness:species *df* = 6, *p* < 0.001). With respect to larval diet, we found that specialist species were significantly more toxic than generalist species (*nested phylogenetic ANOVA* diet *F* = 106.73, *p* < 0.001, diet:species *F* = 15.63, *p* < 0.001; *nested ANOVA* diet *df* = 1, *p* < 0.001*,* diet:species *df* = 6, *p* < 0.001).

Finally, no correlation was found between warning signal abundance and CN concentration (controlling for phylogeny *t* = −0.868, *p* = 0.418, without phylogeny effect: *df* = 6, *t* = −0.87, *p* = 0.418). However, the most toxic species, *H. sara* and *H. doris*, did display the rarest warning signal (Fig. 2).

**Fig. 4:**
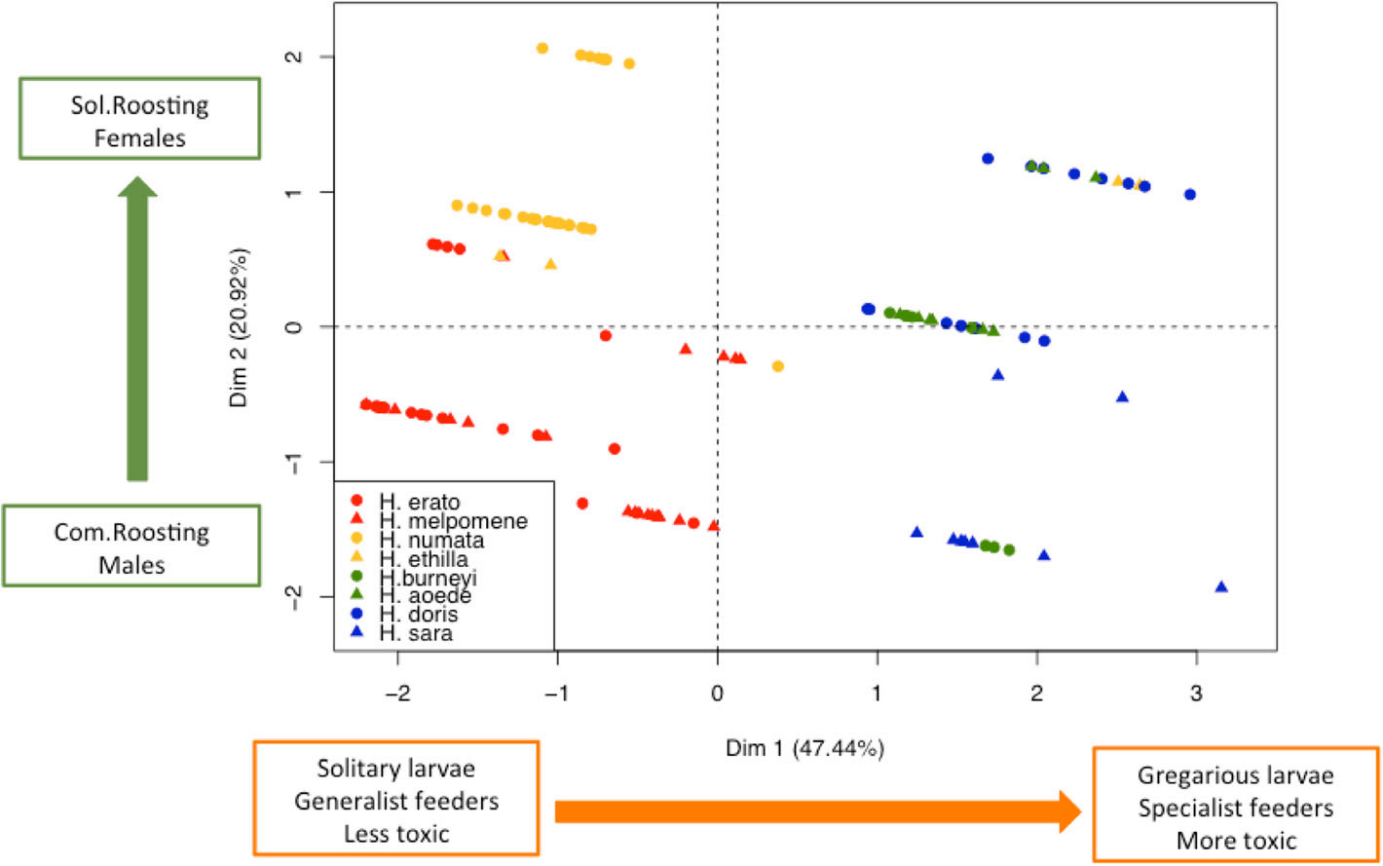
Individuals distributed in a Factor Analysis for Mixed Data (FAMD) plot for the first 2 dimensions. Different colours represent different mimicry rings (red is *postman*, orange is *tiger*, green is *dennis rayed* and blue is *blue/yellow*). Circles represent the individuals of the less toxic species of each pair, while triangles stand for individuals belonging to the most toxic species. The green and orange arrows represent the direction of the variation on the ecological and behavioural traits that we included in our analyses. In squares, the extreme phenotypes for each trait and the area in the plot where they are located

To account for correlations within the different ecological and behavioural traits, and mimicry ring, we performed a Factor Analysis of Mixed Data (FAMD, Fig. 4). Toxicity, larvae gregariousness and specificity of larval diet were correlated, and contributed similarly to the first dimension of the FAMD, explaining 46.67% of the total variation (Additional file 3: Figure S1). Roosting behaviour and sex mostly contributed to the second FAMD dimension that explains 21.89% of the total variation. Less toxic species generally have solitary and generalist larvae, are common rooster as adults and belong to *postman* and *tiger* mimicry rings. In contrast, *blue/yellow* and *dennis rayed* rings contain more toxic species, and have gregarious and specialised larvae, highlighting that both convergent evolution toward similar warning signal and life-history traits might influence toxicity levels.

## Discussion

### Natural interspecific variation

Large variations in inter-specific toxicity were observed in *Heliconius* butterflies caught within the same natural habitat. For instance, *H. sara* individuals were on average 2.25 times more toxic than *H. melpomene* individuals*.* This is comparable to the three-fold difference in concentration previously reported by Engler-Chaouat and Gilbert [20] for the same pair of species when individuals were reared on their preferred host plant. However, toxin concentrations reported by [20] showing that *H. melpomene* (0.719 ± 0.072 μg CN/dwmg) was more toxic than *H. numata* (0.509 ± 0.055 μg CN/mg) and *H. doris* (0.357 ± 0.029 μg CN/mg) contrast with our findings, in which *H. numata* (2.241 ± 0.74 μg CN/mg) and *H. melpomene* (2.601 ± 0.96 μg CN/mg) showed lower total CN concentrations than *H. doris* (4.183 ± 1.43 μg CN/mg). This discrepancy may stem from the cyanide hydrolysis methods applied. Engler-Chaouat and Gilbert [20] used the substrate specific *β-glucosidase* enzyme originally present in the butterfly samples, to hydrolyse linamarin, one of several cyanogenic compounds found in *Heliconius* butterflies [52]. In our study, we used a non-selective method under strong acidic conditions that enables chemical hydrolysis of all cyanogenic compounds carried by our analysed butterflies, irrespective of chemical structure. This also explains why the concentrations that we report are larger than those reported by [20]. The differences between the two studies thus highlight how species not only contain different cyanide concentrations, but also cyanogenic glucosides with different structures, and possibly different enzymes able to hydrolyse each cyanogenic compound. These quantitative and qualitative differences may produce variation in predators’ rejection behaviour and merit further investigation, since predator responses to these variations ultimately shape selective pressure acting on the evolution of mimicry.

### Does mimicry explain the differences in cyanide concentration? Within and between mimicry ring variation

Mimicry rings have significantly different toxicity levels, even after accounting for variation in toxicity among species within mimicry rings. This held true when phylogenetic correction was applied, showing that relatedness among co-mimics does not explain similarity in level of toxicity. However, species sharing a common warning signal generally exhibited similar ecologies and behaviours. For instance ecological convergence in flight height [53] and microhabitat use [53, 54] have been found among co-mimics. Here, we report how life-history and behavioural traits are strongly associated with cyanide concentration (Fig. 4). Despite our limited sample size, most ecological characters tested (except abundance) were described as binomial variables and were evenly represented in our dataset (usually 4:4). Furthermore, our comparative analyses accounted for intraspecific variation on more than 100 specimens. However, increasing the number of species tested within rings would allow to estimate which of these different life history and ecological traits coevolve with toxicity, or whether convergence in one drives convergence in others.

### Within species variations in levels of chemical defence: sex differences

Females of mimetic and non-mimetic species have been reported to suffer more attacks than males [55]. Such differences may result from a generally less agile flight (perhaps due to heavier, egg-laden bodies), and also from slower, more predictable flying when searching for host-plants on which to oviposit [56]. Greater vulnerability has likely promoted increased protection in females, such as higher CN concentration, or the female-limited mimicry observed in certain Batesian mimetic butterflies [55]. In *Heliconius,* males transfer a spermatophore to the females that is rich in cyanide compounds in addition to containing sperm [35]. Given that wild caught females and males are generally mated, we expected to find higher cyanide concentration in females. Only females of *H. doris* showed higher cyanide concentrations than males. The overall lack of cyanide concentration differences between sexes for the other *Heliconius* species included in our study suggest that despite the transfer of spermatophores, males and females are similarly defended.

### Ecological factors influencing levels of chemical defence in mimetic species

#### Abundance

Predators’ ability to learn and associate warning signals with chemical defences is strongly influenced by prey features such as unpalatability and abundance (i.e., how often predators encounter a warning signal). Predators learn the association between a given warning signal and its unprofitability faster when the aversive stimulus is stronger [57]. However, although the *blue/yellow* colour pattern was the most toxic and also the least abundant mimicry ring, we were unable to detect a trend between abundance and toxicity across the four mimicry rings. We only included two species per mimicry ring for our toxicity analyses, a non-representative sampling from mimicry rings that can comprise dozens of species. The *tiger* ring, for example, includes at least 89 species living in sympatry (Fig. 2). Moreover, current toxicity presumably reflects past selective (and historical) processes, that may well differ from current selective processes. Our abundance estimation is limited to the last five years sampling and might not be representative to the historical abundances of the different mimicry rings in the area. This could also explain the general lack of correlation between abundance and toxicity.

We found high variation in toxicity both within and between co-mimetic species. We detected a possible case of automimicry (i.e., we detected no CN in two *H. erato* individuals), as well as significant differences between three of the four pairs of co-mimics (Fig. 3). When unpalatability variation can be detected within a mimicry ring (either within species and/or between co-mimics), predators learn to sample it more carefully, to accurately determine the real unpalatability value of each prey item (the “go-slow strategy”) [58], instead of being totally deterred by a given warning signal [59]. Such variation thus reduces the efficiency of warning signal, because it increases the predator-sampling effect on the mimicry ring. Larger abundances might therefore be associated with larger toxicity variation, because the per capita risk of predation is reduced in abundant mimicry rings. Whether variability in toxicity is driven by variation in abundance remains nevertheless unclear.

### Selection by predators at larval and adult stages

The two most toxic species *H. sara* (*blue/yellow* mimicry ring), and *H. aoede* (*dennis-rayed* mimicry ring) are specialist feeders, probably due to specific metabolic pathways adapted to the biochemical composition of the host-plant [20]. But they also belong to the two least abundant mimicry rings, showing the association between mechanisms that confer higher toxicity, displayed by species exhibiting a warning signal at low abundance. In this way, the few encounters between predator and prey, given their low abundance, will be highly memorable, given their high toxicity, in contrast to more palatable prey that will be more sampled before being learned as unprofitable [60]. Both high specialization and low abundance of these mimicry rings, seem strongly associated with higher toxicity.

We also found a positive correlation between larval gregariousness and toxicity. Larval mortality can reach 95% on first instars and includes predation, parasitism and desiccation among other factors [61]. Although they all have scoli (spine-like structures), *Heliconius* larvae are mostly gregarious and rather cryptic for highly toxic species (Fig. 1). Gregariousness can reduce per capita detectability [62] and predation by the “dilution effect” [63], because predators will not eat all larvae due to predator satiation and/or time spent feeding on them [64]. However, parasite transmission is usually density-dependent [65], thus gregariousness might increase vulnerability to parasites. The evolution of mechanisms conferring higher toxicity could thus have evolved in response to selection exerted by parasites and parasitoids. Gregariousness has also been reported as a mechanism to accumulate higher energy resources by larvae of the African armyworm *Spodoptera exempta* [66]. On a protein deficient diet, gregarious larvae of *S. exempta* accumulated more body nitrogen per amount consumed, in contrast to solitary larvae [67]. Whether it can also increase the CN intake or its synthesis by larvae remains an open question.

Contrary to the findings for larvae, at the adult stage, communal roosting was associated with lower chemical protection. Communal roosting of aposematic individuals has been shown to enhance warning signal conspicuousness [68], and can therefore limit predation pressure. This might explain a relaxation of selection for high levels of defence in aposematic species with gregarious behaviour as adults. Evolution of chemical defence therefore seems to be shaped by predation specific pressure exerted both at larval and adult stages.

### Mimicry, ecological convergence and defences variation

According to our results, chemical defences variation is associated to warning signal convergence, but is not the only trait that explains differences in protection level. Larval diet specialization and gregarious behaviour in larvae and in adults are also associated to distinct levels of cyanide concentration in *Heliconius*. It is possible that mimicry has promoted convergence in ecology and behaviour that at the end produced similar defence levels between co-mimics. But is also possible that mimicry is an outcome of having similar protection levels [13], behaving similarly and exploiting resources in a similar way. Whether these traits are evolving one after the other or are coevolving together, remains an open and interesting question to solve.

### Effects of chemical defence on predators’ behaviour

The correlation between CN concentration and repulsive behaviour is not straightforward. Arias et al. [69] conducted taste-rejection experiments towards different *Heliconius* species with naïve predators, removing birds’ access to any visual and odour cues. Based on taste only, tested birds rejected *H. numata*, *H. erato* and *H. melpomene* similarly, ingesting a limited fraction of them, regardless of the contrasting cyanide concentrations detected in our study for these different species [69]. This implies that even small concentrations of cyanide can produce aversive reactions in predators. However, behavioural experiments studying the possible effects after consumption, probably involved in the learning of a warning signal as aposematic, might show that minute differences in cyanide concentration are relevant. Short- and long-time effects of chemicals on birds are important to test given that some natural predators have been reported to feed on *Heliconius* butterflies, such as tropical kingbirds (*Tyrannus melancholicus*) [70]. Furthermore, long-time dose effects of chemicals have been predicted to affect mimicry dynamics by determining the number of attacks needed for predators to learn and avoid a given warning signal [71, 72].

The effect of specific cyanide compounds or other toxins present in the butterflies was not explored in our study but deserves consideration regarding predator behaviour. For instance, pyrazines are responsible for some of the strongest butterfly odours, and are probably involved in predation learning [16], and have been reported in *Heliconius* species such as *H. melpomene* [73]. Additionally, other undetected substances, such as β-carboline alkaloids, have been found in *H. ismenius* [74] and might also be involved in predator learning. Alkaloids may contribute to butterflies’ bitterness and bad taste, in addition to their toxicity [75]. The unpalatability on *Heliconius* butterflies therefore relies on a diversity of chemical compounds, not exclusively on cyanogenic glucosides. Further detailed investigation on the presence of a variety of such chemical defences, and their effects on predators, are required.

## Conclusions

Large variations in CG concentration were found among sympatric *Heliconius* species and within mimicry rings in natural populations. Although this variation is associated to mimicry, our results highlight the importance of other ecological traits and life-history features on the evolution of such variation. Our study thus stresses the need to investigate ecological traits to understand the evolution of toxicity in mimetic species.

## Additional files

Additional file 1: Supplementary methods. (DOC 17 kb)
Additional file 2: Figure S1. Correlation among variables and their relative contributions to the first 2 summary dimensions of the Factor Analysis for Mixed Data (FAMD). Distances between variables are related to their correlation. (DOCX 26 kb)
Additional file 3: Table S1. Species included in abundance estimation of each mimicry ring and their chemical defences: pyrrolizidine alkaloids (PA), cyanogenic glucosides (CG), or unknown (U) (DOC 34 kb)
