# Supplementary material for: Variation in cyanogenic compounds concentration within a Heliconius butterfly community: does mimicry explain everything?
Source: BMC Evol Biol. 2016 Dec 15;16:272. doi: 10.1186/s12862-016-0843-5 (PMC5160018; doi:10.1186/s12862-016-0843-5)
Supplement: Additional file 1: — Supplementary methods. (DOC 17 kb) [file 12862_2016_843_MOESM1_ESM.doc]

Supplementary methods:

N-chlorosuccinimide/succinimide oxidizing reagent preparation

0.25 g of succinimide were dissolved in 40 mL of distilled water. Then, 0.025 g of N-chlorosuccinimide were added and mixed by stirring. The solution was then dissolved up to 100 mL using distilled water.

Barbituric acid/pyridine reagent

2 g of Barbituric acid were dissolved in a small quantity of distilled water and 10 mL of pyridine were added. The solution was diluted to 50 mL with distilled water and stored in the dark in the fridge.
