# Supplementary material for: Variation in cyanogenic compounds concentration within a Heliconius butterfly community: does mimicry explain everything?
Source: BMC Evol Biol. 2016 Dec 15;16:272. doi: 10.1186/s12862-016-0843-5 (PMC5160018; doi:10.1186/s12862-016-0843-5)
Supplement: Additional file 2: Figure S1. — Correlation among variables and their relative contributions to the first 2 summary dimensions of the Factor Analysis for Mixed Data (FAMD). Distances between variables are related to their correlation. (DOCX 26 kb) [file 12862_2016_843_MOESM2_ESM.docx]

**Supplementary 1**. Correlation among variables and their relative contributions to the first 2 summary dimensions of the Factor Analysis for Mixed Data (FAMD). Distances between variables are related to their correlation.
