# Supplementary material for: Variation in cyanogenic compounds concentration within a Heliconius butterfly community: does mimicry explain everything?
Source: BMC Evol Biol. 2016 Dec 15;16:272. doi: 10.1186/s12862-016-0843-5 (PMC5160018; doi:10.1186/s12862-016-0843-5)
Supplement: Additional file 3: Table S1. — Species included in abundance estimation of each mimicry ring and their chemical defences: pyrrolizidine alkaloids (PA), cyanogenic glucosides (CG), or unknown (U) (DOC 34 kb) [file 12862_2016_843_MOESM3_ESM.doc]

**Supplementary material**

**Table 1.** Species included in abundance estimation of each mimicry ring and their chemical defences: pyrrolizidine alkaloids (PA), cyanogenic glucosides (CG),or unknown (U)

| Mimicry ring | Family | Species | # indiv. | PA or CG? |
| --- | --- | --- | --- | --- |
| blue/yellow | Nymphalidae | *Heliconius congener* | 4 | CG |
| blue/yellow | Nymphalidae | *Heliconius doris (blue morph)* | 24 | CG |
| blue/yellow | Nymphalidae | *Heliconius leucadia* | 1 | CG |
| blue/yellow | Nymphalidae | *Heliconius sara* | 51 | CG |
| blue/yellow | Nymphalidae | *Heliconius wallacei* | 16 | CG |
| dennis-rayed | Erebidae | *Chetone? Dennis like* | 1 | PA |
| dennis-rayed | Nymphalidae | *Eueides tales* | 14 | CG |
| dennis-rayed | Nymphalidae | *Heliconius aoede* | 69 | CG |
| dennis-rayed | Nymphalidae | *Heliconius burneyi* | 21 | CG |
| dennis-rayed | Nymphalidae | *Heliconius demeter* | 14 | CG |
| dennis-rayed | Nymphalidae | *Heliconius doris (red morph)* | 2 | CG |
| dennis-rayed | Nymphalidae | *Heliconius elevatus* | 3 | CG |
| dennis-rayed | Nymphalidae | *Heliconius erato emma* | 19 | CG |
| dennis-rayed | Nymphalidae | *Heliconius melp. agalaope* | 22 | CG |
| dennis-rayed | Nymphalidae | *Heliconius xanthocles* | 3 | CG |
| postman | Nymphalidae | *Heliconius erato favorinus* | 147 | CG |
| postman | Nymphalidae | *Heliconius eratosignis* | 24 | CG |
| postman | Nymphalidae | *Heliconius melp. amaryllis* | 100 | CG |
| postman | Nymphalidae | *Heliconius timareta* | 19 | CG |
| tiger | Erebidae | *Chetone histriomorpha* | 5 | PA |
| tiger | Erebidae | *Chetone hydra* | 2 | PA |
| tiger | Erebidae | *Chetone? Tiger like* | 2 | PA |
| tiger | Nymphalidae | *Athyrtis mechanitis salvini* | 1 | PA |
| tiger | Nymphalidae | *Callithomia alexirrhoe butes* | 1 | PA |
| tiger | Nymphalidae | *Callithomia hezia phagesia* | 2 | PA |
| tiger | Nymphalidae | *Ceratinia ?* | 1 | PA |
| tiger | Nymphalidae | *Ceratinia neso tarapotis* | 18 | PA |
| tiger | Nymphalidae | *Ceratinia tutia* | 40 | PA |
| tiger | Nymphalidae | *Ceratinia tutia ssp. nov.* | 39 | PA |
| tiger | Nymphalidae | *Dircenna jemina varina* | 1 | PA |
| tiger | Nymphalidae | *Eueides isabella* | 31 | CG |
| tiger | Nymphalidae | *Eueides lampeto carbo* | 1 | CG |
| tiger | Nymphalidae | *Eueides vibilia* | 4 | CG |
| tiger | Nymphalidae | *Forbestra equic. equicoloides* | 2 | PA |
| tiger | Nymphalidae | *Forbestra olivencia* | 2 | PA |
| tiger | Nymphalidae | *Forbestra olivencia olivencia* | 3 | PA |
| tiger | Nymphalidae | *Forbestra proceris* | 4 | PA |
| tiger | Nymphalidae | *Heliconius ethilla aerotome* | 37 | CG |
| tiger | Nymphalidae | *Heliconius hecale felix* | 24 | CG |
| tiger | Nymphalidae | *Heliconius numata* | 6 | CG |
| tiger | Nymphalidae | *Heliconius numata arcuella* | 20 | CG |
| tiger | Nymphalidae | *Hel. num. arc. f. seraphion?* | 1 | CG |
| tiger | Nymphalidae | *Heliconius numata aurora* | 17 | CG |
| tiger | Nymphalidae | *Hel. Num. aurora x elegans(?)* | 1 | CG |
| tiger | Nymphalidae | *Heliconius numata bicoloratus* | 61 | CG |
| tiger | Nymphalidae | *Heliconius numata elegans* | 1 | CG |
| tiger | Nymphalidae | *Heliconius numata illustris* | 7 | CG |
| tiger | Nymphalidae | *Heliconius numata silvana* | 12 | CG |
| tiger | Nymphalidae | *Hel. numata tarapotensis* | 32 | CG |
| tiger | Nymphalidae | *Heliconius numata timaeus* | 1 | CG |
| tiger | Nymphalidae | *Hel. pardalinus sergestus* | 3 | CG |
| tiger | Nymphalidae | *Hyalyris oulita oulita* | 46 | PA |
| tiger | Nymphalidae | *Hyalyris oulita ssp. nov.* | 8 | PA |
| tiger | Nymphalidae | *Hyalyris schlingeri* | 11 | PA |
| tiger | Nymphalidae | *Hyposcada anchiala kezia* | 9 | PA |
| tiger | Nymphalidae | *Hyposcada anchiala mendax* | 8 | PA |
| tiger | Nymphalidae | *Hypothryis ?* | 1 | PA |
| tiger | Nymphalidae | *Hypothyris anastasia anastasia* | 3 | PA |
| tiger | Nymphalidae | *Hypothyris cantobrica* | 1 | PA |
| tiger | Nymphalidae | *Hypothyris cant. cantobrica* | 8 | PA |
| tiger | Nymphalidae | *Hypothyris euclea* | 3 | PA |
| tiger | Nymphalidae | *Hypothyris euclea ssp. nov. 1* | 5 | PA |
| tiger | Nymphalidae | *Hypothyris euclea ssp. nov. 2* | 40 | PA |
| tiger | Nymphalidae | *Hypothyris fluonia pardalina* | 3 | PA |
| tiger | Nymphalidae | *Hypothyris fluonia uchiza* | 1 | PA |
| tiger | Nymphalidae | *Hypothyris man. mansuetus* | 28 | PA |
| tiger | Nymphalidae | *Hypothyris man. meterus* | 20 | PA |
| tiger | Nymphalidae | *Hypothyris ninonia antonina* | 29 | PA |
| tiger | Nymphalidae | *Hypothyris ninonia ssp. nov. 1* | 4 | PA |
| tiger | Nymphalidae | *Hypothyris ninonia?* | 10 | PA |
| tiger | Nymphalidae | *Hypothyris sp* | 6 | PA |
| tiger | Nymphalidae | *Hypothyris sp. nov.* | 2 | PA |
| tiger | Nymphalidae | *Lycorea cleobea* | 2 | PA |
| tiger | Nymphalidae | *Lycorea halia ?* | 2 | PA |
| tiger | Nymphalidae | *Mechanitis lysimnia (x polymnia)* | 1 | PA |
| tiger | Nymphalidae | *Mechanitis lysimnia roqueensis* | 62 | PA |
| tiger | Nymphalidae | *Mechanitis mazaeus* | 6 | PA |
| tiger | Nymphalidae | *Mechanitis mazaeus ?* | 10 | PA |
| tiger | Nymphalidae | *Mechanitis mazaeus deceptus* | 38 | PA |
| tiger | Nymphalidae | *Mechanitis mazaeus deceptus x ?* | 2 | PA |
| tiger | Nymphalidae | *Mech. maz. deceptus x plagifera?* | 1 | PA |
| tiger | Nymphalidae | *Mechanitis mazaeus fallax* | 9 | PA |
| tiger | Nymphalidae | *Mech. mazaeus/messenoides* | 1 | PA |
| tiger | Nymphalidae | *Mechanitis messenoides* | 8 | PA |
| tiger | Nymphalidae | *Mech. messenoides deceptus* | 3 | PA |
| tiger | Nymphalidae | *Mechanitis polymnia* | 48 | PA |
| tiger | Nymphalidae | *Mechanitis polymnia dorissides* | 13 | PA |
| tiger | Nymphalidae | *Mech. polymnia proceriformis* | 110 | PA |
| tiger | Nymphalidae | *Mechanitis sp* | 2 | PA |
| tiger | Nymphalidae | *Melinaea isocomma simulator* | 1 | PA |
| tiger | Nymphalidae | *Melinaea ludovica ludovica* | 2 | PA |
| tiger | Nymphalidae | *Melinaea marsaeus mothone* | 26 | PA |
| tiger | Nymphalidae | *Melinaea marsaeus mothone* | 5 | PA |
| tiger | Nymphalidae | *Melinaea marsaeus phasiana* | 26 | PA |
| tiger | Nymphalidae | *Melinaea marsaeus rileyi* | 2 | PA |
| tiger | Nymphalidae | *Melinaea marseus phasiana* | 1 | PA |
| tiger | Nymphalidae | *Melinaea menophilus hicetas* | 3 | PA |
| tiger | Nymphalidae | *Melinaea menophilus ssp. nov. 1* | 15 | PA |
| tiger | Nymphalidae | *Melinaea satevis cydon* | 2 | PA |
| tiger | Nymphalidae | *Melinaea satevis tarapotensis* | 6 | PA |
| tiger | Nymphalidae | *Napeogenes duessa duessa* | 3 | PA |
| tiger | Nymphalidae | *Napeogenes larina otaxes* | 5 | PA |
| tiger | Nymphalidae | *Napoegenes larina* | 1 | PA |
| tiger | Nymphalidae | *Thyridia psidii ino* | 2 | PA |
| tiger | Nymphalidae | *Tithorea harmonia* | 10 | PA |
| tiger | Nymphalidae | *Tithorea harmonia martina* | 13 | PA |
| tiger | Nymphalidae | *Tithorea tarricina bonita* | 1 | PA |
| tiger | Riodinidae | *Stalachtis calliope (mimic)* | 1 | U |
